# The role of evolutionary game theory in spatial and non-spatial models of the survival of cooperation in cancer: a review

**DOI:** 10.1098/rsif.2022.0346

**Published:** 2022-08-17

**Authors:** Helena Coggan, Karen M. Page

**Affiliations:** Department of Mathematics, University College London, London, UK

**Keywords:** evolutionary game theory, cancer modelling, evolutionary graph theory, games on epithelia

## Abstract

Evolutionary game theory (EGT) is a branch of mathematics which considers populations of individuals interacting with each other to receive pay-offs. An individual’s pay-off is dependent on the strategy of its opponent(s) as well as on its own, and the higher its pay-off, the higher its reproductive fitness. Its offspring generally inherit its interaction strategy, subject to random mutation. Over time, the composition of the population shifts as different strategies spread or are driven extinct. In the last 25 years there has been a flood of interest in applying EGT to cancer modelling, with the aim of explaining how cancerous mutations spread through healthy tissue and how intercellular cooperation persists in tumour-cell populations. This review traces this body of work from theoretical analyses of well-mixed infinite populations through to more realistic spatial models of the development of cooperation between epithelial cells. We also consider work in which EGT has been used to make experimental predictions about the evolution of cancer, and discuss work that remains to be done before EGT can make large-scale contributions to clinical treatment and patient outcomes.

## 1. Introduction

Cancer is an inherently evolutionary process [1]. In their seminal 2000 paper, Hanahan & Weinberg [2] set out six characteristics shared by all human cancers, which are acquired by a process of successive genetic mutation and eventually result in populations of cells with sufficiently altered physiology to be termed cancerous. Mutations can occur on a variety of scales, from single altered nucleotides (point mutations) to entire missing or additional chromosomes. The vast number of cells in the human body is such that any conceivable mutation is statistically likely to exist in at least one cell [3], yet cancer cells are remarkably consistent in their core mutations. Often every neoplastic cell within a large tumour will contain the same baseline ‘driver’ set of genetic alterations, which occur early on in tumour growth [4], and half of all early-cancer mutations occur within just nine genes [5].

Why, then, are these particular mutations so successful? To answer that question, we must consider the factors that give a mutation-bearing cell an advantage over a resident population in competition for space and resources, allowing it to ‘out-reproduce’ its neighbours and take over the population. This is complicated by the fact that some mutations confer a reproductive advantage in certain environments but not in others. West *et al.* [6] have suggested that spatial constraints are crucial to the developmental trajectory of invasive cancers, with otherwise neutral mutations selected for or against depending on the environment in which they first occur. Gatenby *et al*. [7] have argued that the traditional distinction between ‘driver’ (evolutionarily beneficial) and ‘passenger’ (evolutionarily neutral) mutations is inappropriate without knowing their ecological and environmental context, and that the six hallmarks of cancer can likely be acquired through a variety of genetic trajectories. There is also significant evidence for genetic heterogeneity *within* cancerous populations [8], fuelled by neutral mutations which occur later in tumour growth and allow the tumour to quickly adapt to changes in environment. However, this heterogeneity is a double-edged sword: carrying too many passenger mutations has been shown to reduce tumour growth in a mouse model of human cell lines [9].

The reproductive advantage conferred by a particular mutation, therefore, is clearly not fixed, and depends on a cell’s environment and the number of other mutations in its carrier cells. The presence or absence of other cells, and the phenotypic strategies they employ, also impact a carrier cell’s reproductive capacity. The most obvious example of this is contact inhibition (see [10] for a summary), where cells stop proliferating when they are packed too densely, *except* when they are transformed, at which point they begin to overproliferate [11]. Another is the Warburg effect [12], where a population of cells switch to the less-efficient strategy of glycolytic respiration, which acidifies the background environment and damages all non-glycolytic cells. In this context, a cell’s fitness is dependent not just on its own phenotypic strategy (i.e. whether it is glycolytic or not) but on the phenotypic strategies of its neighbours. This is where the framework of evolutionary game theory (EGT) becomes useful: instead of a fixed-fitness model, where the effect of a mutation is considered independently of its context, EGT considers the interplay of different phenotypic strategies within a population in order to develop a fuller accounting of intercellular dynamics.

The purpose of this review is to summarize the contribution of EGT to untangling the mechanisms which allow cancerous mutations to spread, particularly on epithelia, where many common cancers begin [13]. Simple epithelia are thin monolayers of cells kept in strict homeostasis in adult tissue [14], at a roughly constant population size where each cell has around six neighbours [15]. The gradual acquisition of cancerous mutations is very difficult to monitor clinically, since by the time a cancer patient reaches a doctor’s office the process of tumour development is often significantly further advanced. Studies of realistic cancer progression must also contend with the difficulty of tracking exactly which genetic mutations are passed on with each cellular reproduction. This is therefore a context in which the tools of mathematical modelling can be brought to bear, allowing us to make predictions about processes that cannot be witnessed directly (see [16] for an excellent review of the much broader field of mathematical oncology). These ideas have also been used to model linguistic and cultural evolution [17], which take place on a different scale but which are fundamentally also sums of small, difficult-to-record individual events, which we can only hope to understand in terms of the aggregated forces driving them.

The applications of EGT to cancer have been recently well-reviewed by Wölfl *et al.* [18], but the focus of that work is on higher-level phenomena. A great deal of time is spent on a description of cancer treatment as a ‘game’ between the physician and the disease (see also [19] for a thorough discussion of this), but the role of space is considered only briefly, and finite-population and nonlinear-benefit effects only in passing. All of these phenomena must be considered in detail before EGT can be used to understand cancer on the level of interactions between individual cells, which are particularly crucial in the early stages of cancer development, when tumour-cell populations are small. This leaves a gap in the literature which we hope to fill with this review.

Our particular focus is the question of how cooperation develops and survives in tumour populations. Cooperation is crucial to the development of cancer. One of its oft-cited hallmarks is growth-factor self-sufficiency, as noted by Hanahan & Weinberg [2]: cancer cells acquire the ability to produce the substances that indicate to themselves and each other that they can reproduce, instead of waiting for the usual signals from the rest of the body. These growth factors are diffusive, and require some energy cost to generate, so that a cell which produces them increases the reproductive ability of its neighbours while decreasing its own. This system is thus perfectly set up for a ‘tragedy of the commons’ [20]. A ‘defector’ cancer cell, with a mutation allowing it to free-ride off the growth factor produced by its neighbours without producing any itself, should always have a reproductive advantage over growth-factor-producing ‘cooperators’. Its descendants should take over the population and drive cooperators extinct, limiting growth factor production and preventing tumour development. Yet cooperation survives in cancer, not just in this context but in a variety of others. Tumour cells secrete factors that recruit stroma, such as fibroblasts and immune cells, to the cause of cancer development [21]. Producing these substances collectively benefits the tumour population but comes at an individual cost to reproductive fitness. Axelrod *et al.* [22] have suggested that populations which have acquired some of the hallmarks of cancer but not others cooperate to propel each other down the path to tumorigenesis, each producing a growth factor the other needs. They term this phenomenon ‘by-product mutualism’, though it may also be thought of as a division of labour. They also suggest intra-tumoural heterogeneity as evidence for more general forms of cooperation within cancer, with different cell populations paying a fitness cost to develop abilities that the tumour as a whole needs to survive. Cooperation is of course not the only manner in which tumour cells interact: one of the very few studies in which evolutionary-game-theoretic interactions have been quantified experimentally in *in vitro* cancer-cell populations [23] found comensalistic relationships. But we would argue that cooperation is the most promising avenue for theoretical and experimental exploration, since it allows multiple cancer-cell populations to speed each other along the path to malignancy. Identifying the mechanisms by which cooperation develops, and how those mechanisms might be disabled, is a vital step in slowing the development of cancer before metastasis—or even tumorigenesis—can occur.

The outline of this review is as follows. In §3, we discuss some classical matrix games and outline the basic principles of EGT. In §4, we consider the various dynamics that result from these strategy-dependent interactions and efforts that have been made to model them analytically, starting with the well-known base case of the infinite and well-mixed population. Section 5 offers a brief summary of adaptive dynamics, the mathematics that results when evolving traits are modelled as continuous variables. Section 6 discusses the case of finite populations with defined ‘update rules’ governing reproduction. Section 7 focuses on spatial modelling and discusses the advances that have been made over the last two decades in the field of evolutionary graph theory, from theoretical analyses of interacting agents on regular graphs to more advanced simulation-based models of simple epithelia. We consider studies that have applied the ideas in each section to make predictions about the behaviour of cancer, to give the reader a sense of the utility and limitations of EGT. We conclude with a summary of current theoretical challenges within the field, and the experimental and computational work that remains to be done in order to rise to them.

## 2. Games in cancer

The simplest case we consider is that of two agents, each of which can follow strategy A or strategy B (conventionally designated ‘cooperate’ and ‘defect’), interacting to receive a pay-off determined by their *interaction matrix*,

$$\begin{array}{rl} & \begin{array}{cc} \;\;A & B \end{array}\\ & \left(\begin{array}{cc} \alpha & \beta \\ \gamma & \delta \end{array}\right)\begin{array}{c} A\\ B \end{array} \end{array}$$

By convention, this means that a strategy-A individual receives pay-off *α* for interacting with another strategy-A individual and *β* for interacting with a strategy-B individual; the second row describes the pay-offs of strategy-B. Depending on the relative magnitude of pay-offs, this situation leads to various incentive structures, which we refer to as social dilemmas. This is one of many situations in EGT where our terminology is inherited from the origins of game theory in describing human behaviour, and misleadingly implies that individuals must be sentient beings with conscious goals. This is of course not true: in intercellular dynamics, game theory becomes relevant whenever cells behave in a way that affects their own and others’ reproductive fitness, implying the existence of a strategy-dependent pay-off matrix. Here, a *strategy* is simply a phenotype, which may be determined by inheritance (subject to random mutation at birth) or induced as a response to its environment [18]: a cell may produce a growth factor or not, become motile or not, or so on.

The two social dilemmas most often invoked to explain tumour-cell behaviour are the Snowdrift game (also known as the Hawk–Dove game or the game of chicken [24,25] and often studied in yeast [26]) and the Prisoner’s Dilemma [27–29]. The Snowdrift game is defined by the pay-off ranking *γ* > *α* > *β* > *δ* and is relevant to the game of growth factor production if we assume that it is better for a cell to produce a growth factor at some cost to itself, even if no other cells do, than it is to exist in an environment with no growth factor at all.

This is rarely assumed, however, and the better-studied social dilemma for this problem is the Prisoner’s Dilemma, defined by the pay-off ranking *γ* > *α* > *δ* > *β*. This can be made to satisfy the condition of ‘equal gains from switching’, i.e. that *β* + *γ* = *δ* + *α*, which removes one degree of freedom. If we further specify that B-players are not affected by interaction with each other, i.e. *δ* = 0, we can reduce the Prisoner’s Dilemma to the two-parameter form

$$\begin{array}{rl} & \;\;\begin{array}{cc} A & B \end{array}\\ & \left(\begin{array}{cc} b-c & -c\\ b & 0 \end{array}\right)\begin{array}{c} A\\ B \end{array} \end{array}$$

Here cooperation comes at a cost *c* and confers a benefit *b*. A-players are called *cooperators* and B-players are called *defectors*. The best outcome for a player is defecting against a cooperator; the worst is cooperating against a defector.

What kind of scenarios arise from these games? The most famous relevant concept here is that of the strict Nash equilibrium [30], a situation where no player can get a better pay-off by unilaterally changing strategy. The Prisoner’s Dilemma has only one strict Nash equilibrium, where both players defect. We can also consider which strategy is *risk-dominant*, i.e. which strategy is better when no information is known about the other player’s behaviour; here we compare average pay-offs and say that strategy A is risk-dominant if *α* + *β* > *γ* + *δ*. (In the Prisoner’s Dilemma, defection is risk-dominant.) We say that a situation is *Pareto-efficient* if there is no alternative choice of strategies which will leave a player better off without making another worse off. For instance, the all-defection strict Nash equilibrium in the Prisoner’s Dilemma is not Pareto-efficient, because both players would be better off if they both decided to cooperate. A type of two-player game not discussed in detail here is the *coordination game*, defined by *α* > *γ* and *δ* > *β*, i.e. where each strategy is the best play against itself. Neither the Prisoner’s Dilemma nor the Snowdrift game are coordination games, and while there has been some suggestion that coordination games may exist in cancer, they have yet to be explicitly identified [31]. It is worth noting that the oft-quoted one-third rule [32] for the survival of ‘cooperation’—that under certain conditions, cooperation is a favoured strategy if the unstable equilibrium $x_{*}=(\delta -\beta )/(\alpha +\delta -\gamma -\beta )$ is greater than one-third—is only relevant to coordination games.

**Figure 1.**
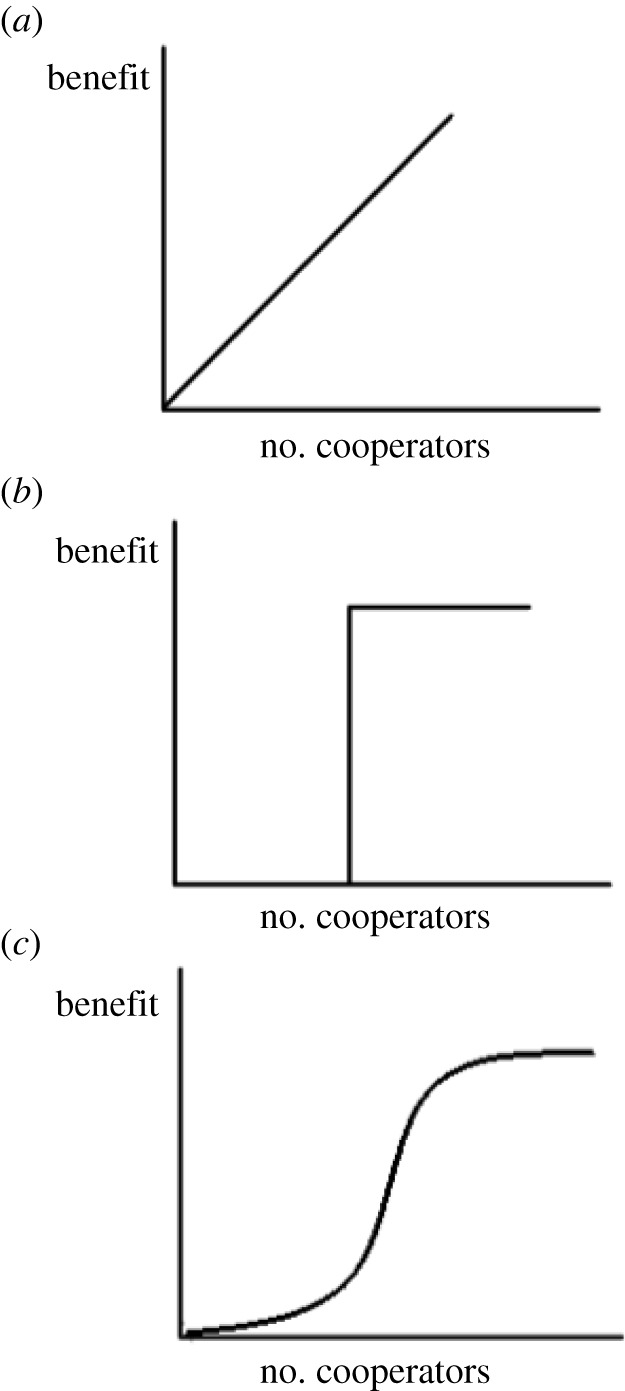
A graphical illustration of the benefit-to-cooperator relationship for (*a*) the N-person Prisoner’s Dilemma, (*b*) a threshold game (e.g. the Volunteer’s Dilemma) and (*c*) a general sigmoid benefit function.

A question that naturally arises is how to extend the two-player game to interactions between larger groups of cancer cells (or indeed any other kind of agent). A common approach is to assume that every agent in a group plays a two-player game with every other, and either accumulates or averages the resulting pay-offs [33]: this is called *pairwise interaction*. Archetti & Scheuring [34] have argued that multiplayer games have fundamentally different dynamics, and cannot be approximated as sums of pairwise games. If we still have only two strategies, A and B, and a group of *N* interacting players, then we can represent a general game with two vectors of length *N* with entries *a*_*k*_ and *b*_*k*_ for *k* = 0, …, *N* − 1, where *a*_*k*_ represents the pay-off to an A-player when it interacts with *k* other A-players and *b*_*k*_ the corresponding pay-off to a B-player interacting with *k* A-players. A wide variety of multiplayer games can be represented in this form [35], the simplest of which is the *N*-person Prisoner’s Dilemma, in which the benefits from cooperators are pooled and shared evenly between all participants, such that *a*_*k*_ = *b*(*k* + 1)/*N* − *c* and *b*_*k*_ = *bk*/*N*. This assumes that the benefit from cooperation is a *linear public good* [36], which is to say it is non-excludable (no individual can prevent another from using it) and increases linearly with the number of cooperators [34]. However, as of 2012, no *in vivo* linear public goods had been observed experimentally, and to the best of our knowledge none have been discovered since. Instead, it has been suggested that the benefit from public goods in cancer is *sigmoidal* in the number of cooperators in a group [37], with pay-offs

$$b_{k}=\frac{V(k)-V(0)}{V(N-1)-V(0)};\;a_{k}=b_{k+1}-c,$$

for a saturating function *V*(*k*) = 1/(1 + e^*s*(*k*/*N*−*h*)^) with inflexion point *h* and steepness *s*. (A graphical illustration of various linear and nonlinear benefit structures is provided in figure 1.) This applies not merely to growth factor production but to cooperation in, for example, the aforementioned Warburg effect [12,38], whereby cancer cells switch to glycolysis for energy production at some energy cost to themselves. This produces lactate, which acidifies the background, suppressing the immune system, encouraging the production of growth factors and promoting the death of non-glycolytic cells [39], which confers a relative reproductive-fitness benefit to glycolytic cells.

A further type of multiplayer game is the threshold public goods game, where some number *K* of cooperators must be present in a group before any benefit is conferred to any player. If *K* = 1 the game is called the Volunteer’s Dilemma [40], relevant to the production of cell–cell adhesion molecules, which provide their benefit to an entire group as soon as one cell within it produces them [41].

## 3. Evolution as a dynamical system

In order to model how the characteristics of a population will change over time, we must make the link between game pay-off and reproductive fitness. Say that a cell interacts with one or more neighbours and obtains a total pay-off *P*. Its reproductive fitness *F* is most commonly modelled as *F* = 1 − *w* + *wP*, where the parameter *w* > 0 is selection strength. In most theoretical analyses, work is done in the limit of weak selection, *w* ≪ 1, which is equivalent to assuming that interactions between cells make only a small difference to their reproductive success. Where *w* = 0, there is no selection; this is referred to as evolution under ‘neutral drift’ [42,43].

The simplest dynamics we can consider are that of an infinitely large, well-mixed population, where every individual is equally likely to interact with every other, and we can neglect self-interactions. If we have two strategies A and B, a general two-player game, and a population of which a fraction *x*(*t*) are A-players, then the expected fitness of an A-player is *F*_A_ = 1 − *w* + *w*(*αx* + *β*(1 − *x*)). If reproduction is proportional to fitness, the fraction of A-players will grow linearly with the fitness advantage of strategy A over the average member of the population, $\dot{x}=x(F_{A}-\bar{F})$. This gives us the replicator equation first written down in [44], and which here is of the form

$$\frac{dx}{dt}=wx(1-x)((\alpha -\gamma )x+(\beta -\delta )(1-x)).$$

This equation has two trivial stationary points, where the population is all-A or all-B, and a heterogeneous equilibrium (which requires *α* > *γ*, *δ* > *β* or *α* < *γ*, *δ* < *β*). As the success of our strategies are dependent on the population composition, we say the population is subject to *frequency-dependent selection*. The replicator equation can also be extended to include mutation, yielding the obviously named replicator–mutator equation [45].

For the Prisoner’s Dilemma, the replicator equation becomes

$$\frac{dx}{dt}=-cwx(1-x).$$

Here, we can see the dynamics we expect from the existence of the all-defector strict Nash equilibrium: there are no heterogeneous equilibria (i.e. cooperators and defectors cannot stably coexist), and the fraction of cooperators in the population, if non-zero, will always decline. If instead players interact in groups of size *n* within the infinite population, then the same mechanics arise. Any individual interacts with *k* cooperators with equal probability for any cooperator fraction *x*, since groups are selected at random. But cooperators always pay an extra cost *c*, and are thus always at a disadvantage. (For a full review of the dynamics of multiplayer two-strategy linear games, see [46].) This leads to the fundamental observation that an infinite well-mixed population interacting in the context of a linear game will drive cooperators to extinction, i.e. *cooperation cannot survive without a mechanism to sustain it* [47]. In order to develop an explanation for the persistence of cooperation in biological systems, we can draw from several possible mechanisms: stochasticity, i.e. the population is finite [32]; assortment, i.e. the population is not well-mixed [48]; and nonlinearity, i.e. the pay-off for interaction is not linear in the number of cooperators [34]. The first two of these mechanisms will be thoroughly discussed below, but it is worth lingering for a moment on the third. The Volunteer’s Dilemma [34] and sigmoid benefit functions [37] have all been shown to support coexistence of cooperation and defection, even in the infinite well-mixed population. For a review of the dynamics of nonlinear games in infinite populations and examples of the above, see [34], the conclusion of which argues lucidly against the overassumption of assortment as an explanation of cooperation where nonlinearity may suffice.

It is worth considering carefully here what we mean by concepts like *equilibrium* and *stability*. Within the framework of replicator dynamics in the infinite population, linearly asymptotic fixed points (i.e. points to which the population will converge if sufficiently close) correspond to strict Nash equilibria [49,50]. Where the fixed point occurs at a cooperator fraction *x*, the strict Nash equilibrium is a mixed strategy where all players cooperate with probability *x*.

These ideas of ‘evolutionary equilibrium’ have be used to glean insight into the population characteristics that cancer cells should acquire over time. This approach was pioneered by Tomlinson 25 years ago [51]. In his original paper, he set up a 3 × 3 pay-off matrix governing the interactions of cytotoxin-producing, cytotoxin-resistant and neutral cells, and found the polyclonal fixed points of the resulting replicator equation (i.e. where the proportions of each type in the population were such that the pay-offs of all three types were equal, and thus their frequencies did not change). Different parameters of the pay-off matrix—for example, those representing the fitness cost of resistance or the impact of cytotoxin on non-resistant cells—were varied to find their impact on the composition of the population at these fixed points. For some regimes, all three types could stably coexist, and for others a single phenotype would become dominant. His crucial observation was that strategies such as cytotoxin production could find evolutionary success even at the expense of the tumour as a whole, and thus that intra-tumour competition could be leveraged by clinicians to impede tumour growth.

Replicator-dynamics models like the above have been explored by Basanta and colleagues, among others, over the last decade and a half, with linear well-mixed EGT models applied to glioma progression [52], prostate cancer [53], the Warburg effect [54], multiple myeloma [55], and to predict the effect of anti-cancer therapies such as the ‘double bind’ of radiotherapy and the p53 vaccine [56] or antiglycolysis treatment [57]. (The replicator-dynamics framework is appropriate when modelling systems where birth and death rates are similar [58], but not for a substantially growing population such as a metastatic tumour.) More recently, Li & Thirumalai [59] extended this technique to nonlinear systems, modelling growth as a saturating function of absorbed benefit (i.e. using $\frac{dx}{dt}=xg(x)$ for *g*(*x*) nonlinear) and comparing with the results of *in vitro* experiments to explain the coexistence of cooperators (IGF-II producers) and defectors (non-producers) in glioblastomas. The usual course of such studies is to construct a simple model of phenotype interactions in the form of a pay-off matrix, and then analyse the dynamics of the resulting infinite-population replicator equation to find which strategies will dominate, coexist, or be driven extinct. The conceptual simplicity of this approach comes at a cost, however: it is assumed that the system can always reach equilibrium, which may not be feasible (a catch identified by Tomlinson in 1997); and in order to keep the analysis of the system’s fixed points algebraically tractable, the pay-off matrix is reduced to two or three parameters, often with significant loss of generality. The imprecise biological meaning of these parameters, which represent the slightly vague concept of ‘fitness costs’ without specifying a mechanism by which they are incurred, makes these assumptions difficult to test experimentally. The actual measurement of pay-off matrix elements requires the construction of an ‘evolutionary game assay’, wherein the proportions of different phenotypes in a culture are varied in order to measure their effects on each other’s replication rates. This is very difficult to do beyond a simple two-species system in a well-mixed environment, much less in a setting mimicking the genetically and spatially heterogeneous environment of a developing tumour [60]. As mentioned, Kaznatzcheev *et al.* [23] found comensalistic relationships between drug-resistant and drug-sensitive cells in an *in vitro* study of non-small-cell lung cancer exposed to alectinib. Similarly, Wu *et al*. [61] fit a pay-off matrix to a system where multiple myeloma cells interacted with bone-marrow stromal cells with a spatially varying concentration of doxorubicin, and found that if the matrix coefficients were made to be functions of drug concentration, the model could successfully predict the evolutionary trajectory of the system. Perhaps most promisingly from a clinical perspective, Athreya *et al*. [62] were able to distinguish between healthy and cancerous lung biopsy samples with an accuracy of 95 per cent by finding the Nash equilibria of a data-derived bimatrix game representing cell–cell interactions in a developing adenocarcinoma. This work, at the interface of machine learning and game theory, shows the potential of even this simple formulation of evolutionary incentives in the diagnosis and treatment of cancer.

## 4. Continuous trait evolution and adaptive dynamics

If strategies are not cleanly partitioned into ‘cooperation’ and ‘defection’, or similar, the concept of an evolutionary equilibrium becomes trickier. For some insight, we turn to the field of adaptive dynamics, which considers strategies or traits as continuous variables determining fitness. A common approach to visualizing this concept is the ‘fitness landscape’, where the fitness of a population is plotted against trait values [63]. This approach suggests an easy way to visualize fitness peaks, but in practice, the idea becomes extremely unwieldy whenever the system has more than two relevant traits or where we have frequency dependence (requiring the landscape to ‘heave and bulge’ as the population moves across it). A more versatile tool is the canonical equation of adaptive dynamics [64], which states that traits evolve continuously according to a general fitness function *F*(*u*, *U*) for trait values *u* of an invasive strategy and *U* of a resident strategy. In two-species form, we have

$$\frac{dU}{dt}=k(u,U){\frac{\partial F(u,U)}{\partial u}}_{u=U}.$$

Here, the quantity *k*(*u*, *U*) is a coefficient scaling selection strength, which can incorporate the effects of mutation and (in a finite population) stochasticity. The dynamics here are intuitive: traits evolve towards fitness maxima, just as physical objects in energy landscapes move towards their minima. This framework gives us a stronger foothold for understanding the concept of evolutionary stability. An evolutionarily stable strategy (ESS) U*, if adopted by most of the population, cannot be replaced by any invasive strategy *u* [65]. This is equivalent to requiring *F*(*u*, U*) < *F*(U*, U*), or *F*(*u*, U*) = *F*(U*, U*) and *F*(*u*, *u*) < *F*(U*, U*) [49,66], so that the invading population is disadvantaged when interacting with the initially dominant resident population and never has a chance to grow. In the linear fitness function represented by our pay-off matrix, this condition is *a* > *c,* or *a* = *c* and *a* > *d*.

A convergence stable strategy (CSS) is such that any strategy *U* in its vicinity will be replaced by a strategy *U* + *ε* slightly closer to it [67]; this is equivalent to requiring that the CSS is an evolutionary attractor, and nearby strategies will evolve towards it. Apaloo [68] introduced the further concept of a neighbourhood invader strategy, which as its name suggests can *invade* any nearby strategy; see [69] for a thorough discussion of the way these properties interact and combine.

Adaptive dynamics can be applied to models of multi-phenotype systems in cancer, using equations of the general form

$$\frac{dx_{i}}{dt}=x_{i}G(x,u_{i},u,R(x))$$

and

$$\frac{du_{i}}{dt}=k(u_{i},u){\frac{\partial G(x,v,u,R(x))}{\partial v}}_{v=u_{i}},$$

where here the fitness function (sometimes called the G-function) is dependent on the population-density vector **x** = (*x*_1_, *x*_2_, …, *x*_*n*_); the strategy vector **u** = (*u*_1_, *u*_2_, …, *u*_*n*_), where *u*_*i*_ is the continuous-strategy variable of the *i*th species; and some function *R*(**x**) representing resource availability. These equations are usually constructed according to a model designed by the researchers to mimic a cancerous system and then solved numerically to generate the population-trajectories *x*_*i*_(*t*). The interested reader is directed to the work of Gatenby and co-workers, most notably the 2008 study [70] in which a G-function was constructed to describe the adaptive landscape of epithelial cells during carcinogenesis. This model was able to incorporate nutrient uptake, the Warburg effect and inter-species competition, controlled by the continuous variable *u*_*i*_ assigned to each phenotype. When these equations were solved numerically, the model predicted a realistic simulation of cancer progression. Cunningham *et al.* [71] used a similar model to investigate the evolutionary progression of metastatic cancers as they spread out from their original environment and are forced to adapt to different tissue conditions, and predicted that regardless of their origin, all phenotypes within a given organ should converge to a similar set of characteristics, which should render them susceptible to targeted therapies. The adaptive-dynamics approach has also been used by Gatenby *et al.* to investigate the emergence of resistance to cytotoxic drugs [72], and was explored in more detail in the recent work by Bukkuri & Brown [73], who provided a thorough overview of how to use adaptive dynamics to represent various models of multi-drug resistance. Their paper presented G-function systems where resistance was assumed to confer varying fitness cost, and where degrees of resistance to separate drugs are assumed to be independent, covarying, or mutually exclusive. This latter possibility is referred to as the ‘double-bind’ model—see also [56]—because a population cannot evolve to become resistant to one without increasing its susceptibility to the other. Adaptive dynamics has been used to suggest more specific clinical treatments: in a 2020 paper, Reed *et al.* [74] used a G-function model of independent resistances to different drugs, where resistance was presumed to inflict a *multiplicative* cost on fitness, to suggest patient-specific timings of ‘second-strike’ therapy in sarcomas. Perhaps most clinically promising is the recent development of ‘adaptive therapy’, whereby anti-cancer drugs are applied in carefully timed cyclic bursts, reducing the size of the tumour while maintaining a sizable population of drug-sensitive cells. Between these bursts, the drug-sensitive population recovers to close to its initial levels, keeping the drug-resistant population under control through competition for resources and thus delaying the onset of metastasis. The cost paid by clinician and patient is to relinquish the aim of ‘curing’ the cancer entirely, as opposed to the current best-practice ‘maximum tolerable dose’ (MTD) approach, which aims to kill all cancerous cells before resistance can develop. Adaptive therapy has been the subject of extensive theoretical and computational modelling in the last few years [75–79]. In these studies, it compares favourably to MTD, though its only clinical trial to date [80] involved too few patients to provide conclusive proof of its efficacy [81]. Evolution-inspired therapies are an exciting new avenue of cancer treatment and their potential will hopefully be explored with further clinical trials in coming years.

## 5. Introducing stochasticity

When finite populations are considered a gulf develops between ideas of Nash equilibrium and evolutionary stability, as we now have to deal with the effects of stochastic drift. Nowak *et al*. [32] showed that it is entirely possible for Nash equilibria to be displaced by other strategies in a population of finite size *N* as a result of stochasticity, which can buffet populations away from stability. Any strategy arising in a finite population must eventually either fixate (take over the entire population) or be driven extinct [82], and so it serves us better in this context to talk about a strategy’s *fixation probability*, *ρ*. A population with *i* mutants and *N* − *i* residents will eventually fixate with probability *ρ*_*i*_; if the probability of reaching *i* + 1 or *i* − 1 mutants in the next timestep are $T_{i}^{+}$ and $T_{i}^{-}$, respectively, then the system takes the form of a random walk [83], from which we obtain the fixation probability of a single mutant appearing in a resident population

$$\rho_{1}=\frac{1}{1+\sum_{i=1}^{N-1}\prod_{\;j=1}^{i}\frac{T_{\;j}^{-}}{T_{\;j}^{+}}}.$$

In order to make use of this, we must consider our *update rules* governing reproduction. In a model of a population of fixed size with overlapping generations, like an epithelium, every timestep must involve a birth and a death; this is called a Moran process [84]. Most studies consider selection acting on *birth*—to our knowledge there have been no analytical studies of the mechanics of Moran processes with selection on death. In ecological models, there are many possible update rules (including Fermi updating [50,82], where two randomly chosen individuals adopt each other’s strategies or not based on a pairwise comparison of their fitnesses), but we will here focus on two: birth–death, where an individual is selected to reproduce proportionally to fitness and then an individual is selected to die at random, and death–birth, where those steps occur in reverse order. This apparently trivial distinction can lead to very different dynamics in more complex models; for details, see §7.

The versatility of this framework has led to a variety of interesting mathematical extensions. The continuous process relevant to the infinite population can be obtained from the Markov chain above in certain limits, (e.g. [82,85]). Traulsen *et al*. [50] identified the relevant parameter as *Nw* for selection strength *w*, and found a smooth transition between ideas of stability in infinite populations and the finite-population requirement that an ESS must also be resistant to replacement by random drift. Antal & Scheuring [83] used a similar approach to find that the time taken for a mutant to fixate under certain conditions of stability is of order *NlnN*. There have also been attempts to introduce axiomatic rigour to evolutionary dynamics in, for example [86–88], the latter of which placed the Moran process within the Cannings model of exchangeable reproduction and found general fixation probabilities for all such processes. Wu *et al.* [89] drew symmetries between a variety of evolutionary processes under weak selection, also described in the infinite-population case by Page & Nowak [87], and considered the effect of including terms of order *O*(*w*^2^) and higher. Traulsen *et al.* [82] extended the finite-population system described above to an arbitrary number of strategies and mutation rate, and found a *critical mutation rate* that fundamentally altered the dynamics of the system. The theoretical frontier of EGT, even in non-spatial models, is vast enough to occupy mathematicians for decades to come.

## 6. The role of space

**Figure 2.**
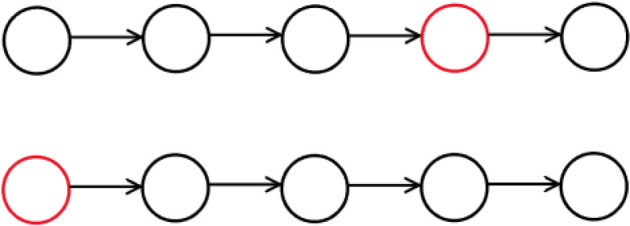
The directed line. Each vertex can reproduce into the vertex to its right. If a mutation appears in the leftmost node (below), it cannot be replaced and must eventually take over the entire line. If it appears in any other node (above) it can never replace the residents to its left and must eventually vanish. The effect of selection is completely negated.

**Figure 3.**
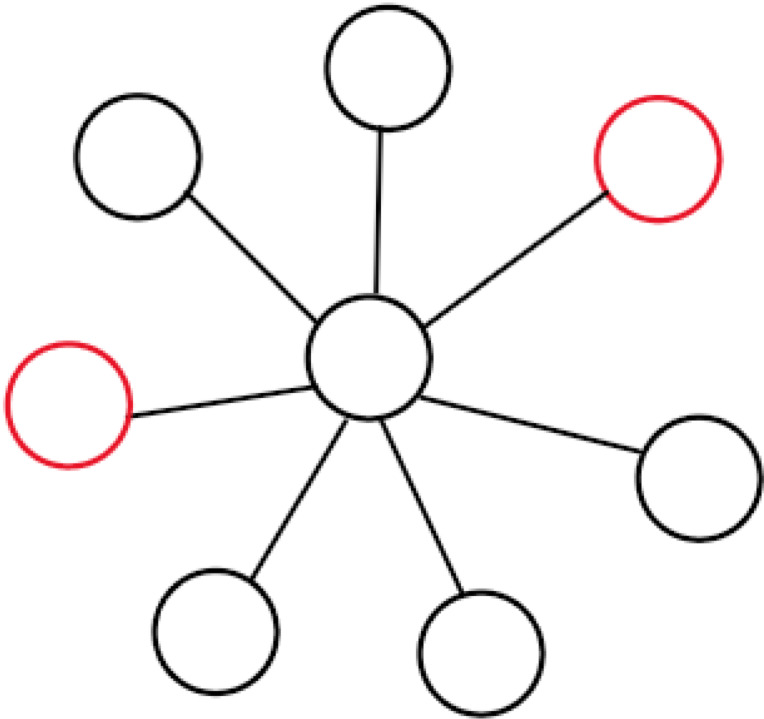
The star, comprised of a collection of peripheral nodes connected to a central node. All connections are undirected; replacement can occur from the periphery into the centre or vice versa. If the number of nodes on the periphery is very large, then the probability of any individual surviving at the centre for long without being replaced is very small, so if a mutation is to spread ‘lastingly’ it must do so periphery-to-periphery, which requires the mutant to be chosen for reproduction twice—once to reproduce into the centre and once again into another peripheral node. The effect of selection is thus ‘squared’.

The results discussed above generally assume a well-mixed population, in which every individual is equally likely to interact with every other. Any realistic model of cancer development must, however, contend with the effects of space [6], as cells are constrained within anatomical structures and are more likely to interact with cells closer to them. These limited interactions create a dependence of fitness on a cell’s location within the tissue architecture, so a mutation’s likelihood of fixation is influenced by where in the tumour it first appears. The interplay of space and survival strategy has been studied within EGT for more than 30 years, first attracting significant attention with Nowak and May’s seminal 1992 paper [90], which illustrated how complex population structures can blossom and die when cooperators and defectors are arranged on a two-dimensional lattice. Progress was made by Lieberman *et al.* [48], who introduced the field of evolutionary graph theory, i.e. the study of evolution on graph-structured populations, in a paper that considered mutants with a constant relative fitness advantage *r*. Two structures discussed in that paper, the directed line and the star (illustrated in figures 2 and 3, respectively), are particularly relevant to the development of cancer and worth lingering on here. Cells arranged in a directed line can only reproduce into the node to their right, and thus a mutation fixates if and only if it arises in the leftmost node, *regardless of its impact on reproductive fitness*; the directed line thus completely suppresses the effects of selection. Colorectal crypts have been experimentally confirmed to follow such one-dimensional neutral drift dynamics [91]. Another interesting structure is the star, which consists of a large number *N* of nodes attached to a single central node. In order for the mutation to spread through the periphery, it must reproduce twice, once into the centre and then again from there into a peripheral node, without being replaced by a resident. This effectively ‘squares’ the effect of selection; a mutation with fitness advantage *r* has fixation probability *ρ*_*WM*_ = (1 − (1/*r*))/(1 − (1/*r*^*N*^)) in the well-mixed population and *ρ*_star_ = (1 − (1/*r*^2^))/(1 − (1/*r*^2*N*^)) in the star structure with birth–death updating. Structures have been found which amplify the effects of selection further still, such as the funnel and the comet [92]. Star-like structures have been suggested to be relevant to pancreatic and colon cancer [6], and may exist in cancers more generally. Tumours have plastic hierarchies which allow stem cells to differentiate and de-differentiate themselves more easily than in normal tissue [93,94]. This ‘star structure effect’ may partly explain the accelerated course of evolution in cancer.

Since this breakthrough, further research has focused on classifying general graph structures in terms of their amplifying or suppressive effects on selection [95,96]. An important distinction to make here is between *directed* graphs, where edges between two vertices *v*_*i*_, *v*_*j*_ are labelled with arrows (e.g. *v*_*i*_ → *v*_*j*_) to indicate that the offspring of *v*_*i*_ may replace the occupants of *v*_*j*_ but not vice versa, and *undirected* graphs, where individuals at any two vertices connected by an edge can replace or be replaced by each other. On a *weighted* graph, connections between vertices are quantified: for every pair of vertices, we assign a probability *w*_*ij*_ that, given that the occupant of *v*_*i*_ is chosen to reproduce, it will replace the occupants of *v*_*j*_. A fundamental result within evolutionary graph theory is the *isothermal theorem* [48], which states that, using birth–death dynamics, a mutant with relative fitness *r* has fixation probability *ρ*_*WM*_ on a graph where the temperature $T_{i}=\sum_{\;j}w_{\;ji}$ of every vertex is equal. (A similar result holds for death–birth dynamics [97].) This condition is called *isothermality*, and indicates that an individual’s probability of replacement—i.e. its mortality—is independent of its position. Such structures never suppress drift. If all vertices have the same *fecundity*, $\sum_{\;j}w_{ij}$, then the graph never suppresses selection. If both conditions are met, the graph is called a *circulation*.

The simplest structure we can use to represent an epithelium is the regular graph, where each node has the same number of neighbours, *k*. Ohtsuki *et al.* [98] studied a version of the Prisoner’s Dilemma on such graphs and found that cooperation is favoured if the cost *per neighbour* *c* and benefit *b* are such that *b*/*c* > *k*. As the graph becomes better-connected, defectors become more likely to encounter cooperators, and thus defection becomes a favoured strategy. The elegant simplicity of this result arises from the conditions under which it was derived, including the assumptions of weak selection and pair approximation [99], meaning that the probability of finding a cooperator or defector at a vertex is assumed to be dependent only on the strategies of its immediate neighbours. As one might expect, more complex systems require more detailed criteria. One interesting aspect of the Ohtsuki result is that it holds only for death–birth updating; for birth–death updating under these conditions, cooperation is never favoured. This result has prompted a great deal of interesting work generalizing update rules and their conduciveness to cooperation, particularly the concept of *compensated relation* as a measurement of the likely genetic impact of helping one’s neighbours [100] and Zukewich’s mixed-update system [101]. In this study, where death–birth updating was used with probability *p* and birth–death otherwise, cooperation was favoured on regular graphs if *b*/*c* > *k*/*p*; thus cooperation can be favoured so long as *p* > 0. There is strong evidence that death precedes birth on homeostatic epithelia [102], so this condition is satisfied for our purposes.

Another useful concept in evolutionary graph theory is the structure factor *σ*, a property of a graph structure (and of its assigned update rule) quantifying the likelihood of cooperators and defectors to assort under weak selection. For the most general form of a two-player game,

$$\begin{array}{rl} & \begin{array}{cc} \;\;A & B \end{array}\\ & \left(\begin{array}{cc} \alpha & \beta \\ \gamma & \delta \end{array}\right)\begin{array}{c} A\\ B \end{array} \end{array}$$

and in the limit of weak selection, strategy A is favoured over strategy B on a graph if *σα* + *β* > *γ* + *σδ* [43]. The higher the level of assortment, *σ*, the more important the self-interaction pay-offs *α* and *δ* become to the probability of fixation. For the Prisoner’s Dilemma, cooperation is favoured if *b*/*c* > (*σ* + 1)/(*σ* − 1). For the well-mixed infinite population, *σ* = 1, and we recover the result that cooperation is never favoured. For the well-mixed finite population of size *N*, *σ* = (*N* − 2)/*N*, which tends to 1 as *N* tends to infinity and the effects of stochastic drift vanish. These results hold for both death–birth and birth–death updating. More complex structures have structure factors that vary by update rule: for instance, a star of size *N* has *σ* = 1 for death–birth updating but *σ* = (*N*^3^ − 4*N*^2^ + 8*N* − 8)/(*N*^3^ − 2*N*^2^ + 8) for birth–death. The former result applies for any mutation rate (i.e. allowing for a certain proportion of random switches in strategy when individuals reproduce), but the latter only holds when mutation is very rare. Results for more complex structures may obey even stricter criteria. Structure factors can be calculated for multiplayer games [96], games with multiple strategies [103] and, for certain systems, by simulating evolution under neutral drift [104]. They have also been used to incorporate the effect of spatial structure into adaptive dynamics [105].

A number of fundamental adjustments need to be made to EGT in order to properly consider the role of space: on a regular graph, for instance, an extra term appears in the replicator equation depending on the pay-off matrix, graph structure and update rule, and this impacts our definition of evolutionary stability [106]. Once we allow the number of neighbours of a vertex to vary slightly, as it does on an epithelium, matters are complicated still further; the criterion for favourability on non-regular graphs is *b*/*c* > 〈*k*^2^ 〉 / 〈 *k* 〉 [107], where <> indicates an average. Cooperation on sparsely connected, regular graphs is also most advantageous where the networks governing interaction (for pay-offs) and competition (for reproduction) overlap [108]. Analytical work on the impact of graph structure has yielded interesting general results for the favourability of cooperation [42], but in order to make specific observations about cellular interactions on epithelia, we must turn to computational research.

Simulation-based work on the impact of graph structure on EGT has traditionally focused on random, scale-free and preferential-attachment graphs [33,109–111] or using imitation-based updating [112], which are relevant to modelling social interactions but inapplicable to epithelia. What results we can use are more or less intuitive: Kun & Scheuring [113], for example, found that allowing vertices to swap positions on a regular graph decreased the favourability of cooperation. This is the effect one might intuitively expect, since random movement disrupts the ability of cooperators to assort. A more realistic model was developed by Pavlogiannis & co-workers [114] in the form of the two-dimensional shift update rule, which allows deaths and births to occur anywhere in the epithelium (known as ‘global updating’) and cells between them to be shifted along a path of least resistance. This approach is very computationally expensive, however, and required several weeks to run on regular grids of maximum size 16×16. Techniques that do not need to aggregate thousands of simulations to calculate probability are significantly cheaper. Shakarian *et al*. [115] have developed a deterministic simulation method for calculating fixation probabilities on any network which tracks the spread of ‘cooperator probability’ without the expense of stochastic simulation.

To more directly study the spread of cooperation on epithelia, researchers have used the Voronoi network, first defined over a century ago, designed to represent the locations of cell nuclei in a realistic biological sample [116,117]. Voronoi nodes have an average of six neighbours and very rarely fewer than four or more than eight. These networks can be simulated dynamically by the inclusion in a computational model of cell walls between nodes, which exert forces on each other and cause the nuclei at their centre to shift around. Archetti [118] simulated a public goods game with sigmoid benefits on a Voronoi network with local updating—i.e. requiring that deaths and births must occur next to each other—and in a model where benefits could diffuse beyond their immediate neighbours, although without realistic fluid dynamics. He found that such networks were slightly less conducive to cooperation than regular networks of the same average degree. He attributed this to two phenomena: a slightly higher average ‘group size’ formed by all nodes touched by the diffusive good, such that defectors were more likely to encounter the benefits of cooperation; and non-uniform cooperator fitness created by variability in neighbour numbers. Studies into the effect of non-uniformity in such systems are ongoing. Kaveh *et al.* [119] and Rychtář & Taylor [120] have found analytically that variations in fitness within an invasive population tend to hinder fixation. However, it has also been suggested [121,122] that *time-dependent* fluctuations in selection actually promote heterogeneity. Mahdipour-Shirayeh *et al.* [123] have also noted that environmental fluctuations can help deleterious mutants fixate on one-dimensional rings, such as those in the colonic crypt.

To better understand the success of cooperation on realistic simple epithelia, Renton & Page studied both the Prisoner’s Dilemma [124] and sigmoid-benefit public goods games [125] on Voronoi tessellations. They found that the crucial mechanism required for cooperation to succeed was the spatial decoupling of birth and death, with nuclei moving under the forces created by cells vanishing and appearing anywhere in the population. This decoupling allowed clusters of cooperators to grow from within, where they are fittest, rather than simply at the boundary, as required by local updating. Once this was implemented, for example, the critical benefit-to-cost ratio required for cooperation to be favoured in the Prisoner’s Dilemma more than halved compared to a static network with death–birth updating. They also found, in accordance with the work of Archetti, that steeper benefit functions enhanced cooperation. By varying their model parameters, they obtained both coexistence and coordination behaviour in their public goods games. Their key result is that cooperation is favoured by local gameplay but global competition for offspring; this will be expanded upon in future work. Cooperation thus becomes more likely when simulations become more realistically biological, which is an enormous relief to scientists trying to explain the existence of experimentally observed phenomena.

Space can also be included in evolutionary-game-theoretic models of cancer without the explicit incorporation of graph structure. Studies such as those discussed in §4, where replicator equations are constructed based on hypothetical pay-off matrices and then examined for their equilibria, can be made ‘pseudo-spatial’ by assuming that cell with one phenotypic strategy have fixed locations and thus can interact more or less with other phenotypes. Flach *et al.* [126] used this approach to construct differential equations describing the interactions between melanoma cells and fibroblasts during tumour growth. In the model, the fibroblasts stabilized ‘free’ cancer cells to become ‘fixed’, and stimulated the division of those ‘fixed’ cells to create ‘blocked’ cells trapped within the tumour. These three types of cancer cell were treated as separate populations, with free/fixed and fibroblast growth rates dependent on population composition as a whole. This pseudospatial approach was also used by Qian *et al.* [127] to build a model of cooperation and defection in niche construction, whereby tumour cells modify their microenvironment to create more amenable conditions for their survival. Other approaches to the inclusion of spatial effects in differential-equation EGT models of cell–cell interactions have been summarised by Durrett & Levin [128,129], including patch models (where the population is segregated into several interacting groups, again without the *explicit* inclusion of space) and reaction–diffusion equations. Their work, along with more recent studies [130–132] suggests that when space is built explicitly into models of cancer, fundamentally different population dynamics are obtained: there is no substitute, as it were, for the reality of space. Agent-based modelling, in which each cell is modelled as an individual and the system is simulated computationally, is built around this concept. In their excellent review, Adami *et al*. [133] provide a compelling case for the use of agent-based modelling in EGT, pointing out that it can easily accommodate stochastic phenomena (probabilistic strategies, large mutation rates) that analytical methods struggle to deal with. An *et al.* [134] also note that agent-based modelling easily implements parallelism, allowing multiple instantiations of the same object (i.e. cells of the same phenotype) to observe the range of their possible behaviours in slightly different conditions. It also allows observations of *emergent behaviour*: group dynamics of interacting populations which cannot be guessed from their behaviour in isolation. However, like all large-scale simulations, agent-based models are generally computationally expensive and time-consuming, and do not inherently *explain* the mechanisms behind their results. They are useful for observing the implications of particular models—An *et al.* describe them as tools for testing ‘thought experiments’—but must be backed up with mathematical verification where at all possible [133]. They can also be used to tease out ‘general phenomena’ from particular models, without attaching precise quantification to those predictions. Manem *et al.* [135], for example, used an agent-based model of individuals in space to find that a mutant with a constant fitness advantage could invade a population more easily from within a population than at its boundary, and with more difficulty on an unstructured mesh than a regular graph. Waclaw *et al.* [4] used an agent-based model of a developing three-dimensional tumour, where successive mutations had multiplicative fitness effects, to emphasize the importance of cell motility to lesion growth and predict that mutations with even a small fitness advantage could spread quickly through cancerous tissue. Such studies can also incorporate cooperation: Komarova incorporated realistic mutation rates into the study of public goods games on two-dimensional regular graphs [136], and found that spatial constraints had complex and varied effects. As with the modelling of any phenomenon, computational simulation is a powerful tool that must be understood as an aid to mechanistic understanding rather than a source of it. With that caution observed, agent-based modelling can be an invaluable tool for the understanding and prediction of cancer.

## 7. Conclusion

The study of EGT and its application to understanding intercellular interactions in cancer has two main purposes. One is to understand the process of tumorigenesis, the process by which life turns against itself, simply because it is fascinating and because it is there. Much of the analytical work summarized in the first half of this review considers cooperation and mutant fixation to that end. The second aim of the field is to improve patient outcomes, often by modelling real-world anti-cancer treatments computationally and analysing clinical data. In a recent study, for example, the idea of destroying the evolutionary advantage of cooperation was experimentally tested by re-engineering and re-injecting extracted cancer cells to allow defectors to take over a population [137].

In order for the study of EGT to achieve its full potential—for those seeking to understand cancer, to treat it, or both—an interdisciplinary effort is required. To make use of studies quantifying precise critical benefit-to-cost ratios required for cooperation to survive on epithelia, for example, we must make precise what we mean by ‘benefit’ or ‘cost’—what biochemicals enforce it, how quickly and how far do they spread, and through what mechanisms can they be altered? If we assume that treatments against public goods raise the point of inflexion *h* of a sigmoid benefit function, as in [118], how quickly does this happen, and by how much? What is the set of public goods (and indeed public detriments, such as lactic acid in the context of the Warburg effect) that influence tumour-cell growth in a given context, and how are these produced and shared between cells? How widespread is the phenomenon of phenotype switching at various stages of tumour development, and between which phenotypes and at what rates does it occur? Dujon *et al.* [138] have an excellent summary of broader questions within the field, collated from 33 experts. Of particular interest to EGT is the question of the extent to which intra-tumoural heterogeneity is a *cause* or *consequence* of oncogenesis, which can partially be addressed experimentally.

Once these questions are answered, a conceptual route can be carved out towards preventative treatments for those at risk of developing epithelial cancers and anti-cancer treatments for those already diagnosed. This will require close collaboration between theoreticians and experimentalists, following the work of Archetti [139] *et al.* and Calbo [140] *et al.*, wherein games were successfully implemented in biological systems. Models more realistic and complex than can be handled by theoretical analysis alone—such as those involving density-dependence of homeostatic replication [141] or contact inhibition and realistic models of cell extrusion [10,142,143]—will require computational research to make predictions and experimental input to test them. Improved methods of monitoring the detailed clinical progression of tumours [18,63] will aid this effort in the coming years. The aim should be a feedback loop between theoretical, computational and experimental researchers, whereby the assumptions of theory are redirected, and new predictions are generated as existing ones are tested by experiment. No single discipline can tackle the problem of halting tumorigenesis without a good grasp of what is known by researchers in other, perhaps philosophically quite distinct, areas of science. Only through collaboration and communication can we make real progress towards understanding and curing cancer.

## Data Availability

The data are provided in the electronic supplementary material [144].
